# Transcription factor NRF2 controls the fate of neural stem cells in the subgranular zone of the hippocampus

**DOI:** 10.1016/j.redox.2017.06.010

**Published:** 2017-06-27

**Authors:** Natalia Robledinos-Antón, Ana I. Rojo, Elisabete Ferreiro, Ángel Núñez, Karl-Heinz Krause, Vincent Jaquet, Antonio Cuadrado

**Affiliations:** a Instituto de Investigaciones Biomédicas “Alberto Sols", Faculty of Medicine, Autonomous University of Madrid (UAM), Centro de Investigación Biomédica en Red sobre Enfermedades Neurodegenerativas (CIBERNED), Madrid, Spain; b Center for Neuroscience and Cell Biology, Institute for Interdisciplinary Research (IIIUC), University of Coimbra, Portugal; c Department of Anatomy, Histology and Neuroscience, Autonomous University of Madrid, Madrid, Spain; d Department of Pathology and Immunology, University of Geneva Medical School, 1 rue Michel Servet, 1211 Geneva, Switzerland

**Keywords:** Hippocampal neurogenesis, Aging, NRF2, Neural stem cells, Subgranular zone, Oxidative stress

## Abstract

Neural stem/progenitor cells (NSPCs) located at the subgranular zone (SGZ) of the hippocampus participate in the maintenance of synaptic networks that ensure cognitive functions during life. Although it is known that this neurogenic niche losses activity with oxidative stress and ageing, the molecular events involved in its regulation are largely unknown. Here, we studied the role of transcription factor Nuclear Factor-Erythroid 2-Related Factor 2 (NRF2) in the control of NSPCs destinies in the SGZ. We first describe that NRF2-knockout (*Nrf2*^-/-^) mice exhibit impaired long term potentiation, a function that requires integrity of the SGZ, therefore suggesting a cognitive deficit that might be linked to hippocampal neurogenesis. Then, we found a reduction in NSCs from birth to adulthood that was exacerbated in *Nrf2*^-/-^ vs. *Nrf2*^+/+^ mice. The clonogenic and proliferative capacity of SGZ-derived NSPCs from newborn and 3-month-old *Nrf2*^*-/-*^ mice was severely reduced as determined in neurosphere cultures. *Nrf2*-deficiency also impaired neuronal differentiation both the SGZ, and in neurosphere differentiation assays, leading to an abnormal production of astrocytes and oligodendrocytes vs. neurons. Rescue of *Nrf2*^*-/-*^ NSPCs by ectopic expression of NRF2 attenuated the alterations in clonogenic, proliferative and differentiating capacity of hippocampal NSPCs. In turn, knockdown of the NRF2 gene in wild type NSPCs reproduced the data obtained with *Nrf2*^*-/-*^ NSPCs. Our findings demonstrate the importance of NRF2 in the maintenance of proper proliferation and differentiation rates of hippocampal NSPCs and suggest that interventions to up-regulate NRF2 might provide a mechanism to preserve the neurogenic functionality of the hippocampus.

## Highlights

• NRF2-knockout mice show impaired hippocampal long term potentiation.
• NRF2-lack impairs the proliferative and clonogenic capacity of neural stem cells.
• The neuronal to glial differentiation ratio is altered in the absence of NRF2.
• NRF2 is required for homeostasis of the hippocampal neurogenic niche.

## Graphical abstract

**Figure:**
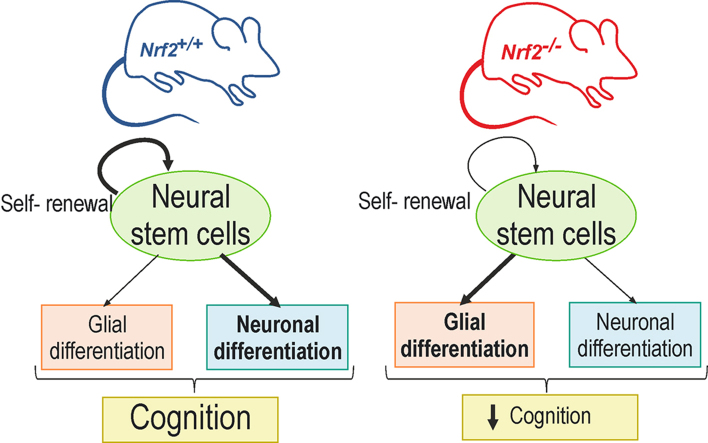

## 1 Introduction

Nuclear Factor-Erythroid 2-Related Factor 2 (NRF2) is a transcription factor that regulates homeostatic responses to multiple stressors. Initially connected with biotransformation reactions, it is known to participate in the regulation of hundreds of human genes involved in antioxidant defense, anti-inflammatory response, autophagy, metabolic reprograming of tumor cells, etc. [1], [2], [3], [4]. Modulation of the expression of these genes provides a global cellular response to promote cell survival, cell growth, self-renewal, differentiation, proliferation, and increased lifespan, which may be relevant to the function of stem cells [5], [6], [7]. NRF2 is expressed in virtually every tissue and promotes survival of stem cells from several origins [8], [9], [10]. In the brain, the direct implication of NRF2 in regulation of neural stem cells (NSCs) has been reported only in connection with the normal decline of its activity during aging and the reduction of the NSCs pool at the subventricular zone (SVZ) of the striatum [11].

The adult brain harbors another region of persistent lifelong neurogenesis: the subgranular zone (SGZ) of the *dentate gyrus* (DG) that has been much less explored [12]. This region contains quiescent neural stem cells (NSCs) that on specific demands are mobilized towards generation of rapidly-dividing neural progenitor cells (NPCs), neurons and astrocytes. Newly generated granular neurons are required for the electrophysiological functioning of the perforant path (PP), involved in spatial learning and memory consolidation [13], [14], [15]. Progressive loss of neurogenesis at the SGZ has been associated with aging, which also correlates with decline of NRF2 activity [16], [17] and with alterations in redox homeostasis. In fact, a relevance of reactive oxygen species (ROS) in neural stem/progenitor cell (NSPCs) physiology is suggested by high expression levels of antioxidant genes encoding SOD1, SOD2, GPX, etc. [18]. However, the role of NRF2 in the homeostasis of the neurogenic niche of the SGZ has not been explored.

In this study, we have analyzed the mobilization of the NSPCs pool of the SGZ in *Nrf2*^-/-^ mice at early age and adulthood. We report that exhaustion of NSCs and NPCs of the SGZ pool occurs more rapidly in *Nrf2*-knockout (*Nrf2*^-/-^) mice, when compared to wild type (*Nrf2*^+/+^) mice, which is correlated with the loss of electrophysiological activity of the perforant path.

## 2 Material and methods

### 2.1 Animals

C57BL/6 mouse colonies of the *Nrf2*^-/-^ and *Nrf2*^+/+^ genotypes were established from funders kindly provided by Prof. Masayuki Yamamoto (Tohoku University Graduate School of Medicine, Sendai, Japan) [19]. All the animal procedures were performed according to the protocols approved by the Ethical Committee for Research of the Universidad Autónoma de Madrid, following institutional, Spanish and European guidelines (Boletín Oficial del Estado of 18 March 1988; and 86/609/EEC, 2010/63/EU European Council Directives).

### 2.2 Electrophysiological recordings

Data were obtained from six urethane-anaesthetized (1.6 g/kg i.p.) mice of 6 months of age per group as described in [20]. Briefly, field potentials were recorded through tungsten macroelectrodes (1 MΩ; World Precision Instruments) stereotaxically implanted in the DG (A: -2,3; L: 2; H: 1.5 mm from bregma, according to the Paxinos and Watson Atlas [21]). Twisted bipolar electrodes for electrical stimulation were aimed at the perforant path (A: -2.5; L: 0.5; H: 1.5 mm from bregma) [22]. Baseline recordings were taken with test stimuli (10–50 mA, 0.3 ms, 0.5 Hz) during 15 min before tetanic stimulation consisting of three pulse trains of 10–50 mA, each pulse lasting 0.3 ms and at 50 Hz. Trains lasted 500 ms and the inter-train interval was 2 s. Recording was maintained for 30 min after tetanic stimulation. Field potentials were 0.1 Hz to 1 kHz band-pass filtered, amplified (P15 Amplifier, Grass Co., USA), and digitized at 10 kHz (CED 1401; Cambridge Electronic Design). Signals analysis was carried out with Spike 2 software (Cambridge Electronic Design, Cambridge, UK). Field potential segments of 5 min were analyzed to obtain the response average. The mean average response during the 15 min period before the tetanic stimulation was considered as 100%. Recordings were accepted for analysis when baseline variability was less than 10%.

### 2.3 Immunofluorescence on mouse tissues and stereological analysis

Brain tissue immunofluorescence was performed in 30 µm-thick sections as previously described [2]. Antibodies are described in Suppl. Table S1. Control sections were treated with identical protocol but with the omission of the primary antibody. Cell counts were performed using Fiji Software (http://fiji.sc/Fiji) [23] in 5 sections of the DG separated by 240 µm.

### 2.4 Cell culture and reagents

Human embryonic kidney HEK293T cells were grown in Dulbecco's modified Eagle's medium (DMEM) (Sigma-Aldrich) supplemented with 10% fetal bovine serum (HyClone), 4 mM L-Glutamine (Gibco) and 80 mg/ml gentamicin (Laboratorios Normon). R,S-sulforaphane (SFN; LKT Laboratories, Inc.). Culture of SGZ-derived NSPCs from new-born 0–4 days old pups was described in [24]. For culture of NSPCs from 3-months-old mice, the same protocol was used with the following medium: DMEM/F12/Glutamax, B27 without vitamin A, 20 ng/ml epidermal growth factor (EGF), 20 ng/ml basic fibroblast growth factor (bFGF), 4 µg/ml heparin, and 100 U/ml penicillin/streptomycin.

### 2.5 Immunofluorescence of neurospheres

Neurospheres were adhered to poly-d-lysine-coated coverslips by centrifugation (634*g*, 5 min), and fixed with 4% paraformaldehyde. Then, neurospheres were washed, blocked in PBS containing 0.5% Triton X-100% and 3% bovine serum albumin and incubated overnight (at 4 °C) with the relevant primary antibodies and for 2 h at RT with the appropriate secondary antibodies coupled to Alexa Fluor 488, 594, or 647 (1:500) (Life Technologies-Molecular Probes, Grand Island, NY, USA). Nuclei were stained with DAPI. Images were quantified using Fiji Software (http://fiji.sc/Fiji).

### 2.6 Serial dilution assay

This assay was performed as described in [11]. Briefly, secondary neurospheres were disaggregated with accutase (StemCell Technologies) and seeded into a 96-well plate at 5, 25, 125, 250, 500 and 1000 cells/well dilution. Cells were kept in 5% CO_2_ and 95% atmospheric air, at 37 °C, in proliferation media, during 7 days, which allowed the formation of tertiary neurospheres. Neurosphere number was determined as de average from 5 wells.

### 2.7 Cell pair assay

As described in [25], a single cell suspension of NSPCs was plated on poly-d-lysine-coated coverslips at a low density (10,000 cells/cm^2^). Cells were cultured for 24 h in DMEM/F12/Glutamax, B27 (without vitamin A) supplemented with 5 ng/ml or 10 ng/ml EGF and 2.5 or 10 ng/ml bFGF, for pup- or adult-derived neurospheres, respectively. After fixation with 4% PFA, cells were immunostained for SOX2. This assay allowed the quantification of symmetric cell divisions (self-renewal with both SOX2^+^ siblings) and asymmetric divisions (differentiation with one SOX2^+^ and one SOX2^-^ sibling).

### 2.8 Differentiation assays and Sholl analysis

Five-day-old secondary neurospheres were plated on poly-d-lysine-coated coverslips and incubated for 7 days in differentiation media (DMEM/F12-Glutamax, B27 without vitamin A, and 100 U/ml penicillin/streptomycin). Immunostaining and quantification were performed as described above. To quantify neuronal differentiation, Sholl analysis was performed using the Simple neurite tracer plugin (http://homepages.inf.ed.ac.uk/s9808248/imagej/tracer/) and the image processing package Fiji (http://pacific.mpicbg.de/wiki/index.php/Main_Page) in images of differentiated neurospheres immunolabeled for DCX (Santa Cruz).

### 2.9 Production of lentiviral stocks and infection of NSPCs

Recombinant lentiviral stocks were produced in HEK 293 T/17 cells by cotransfecting 10 μg of transfer vector (control plasmid, NFE2L2^ΔETGE^ [26], shCO or shNRF2 (shCLN_NM_010902, MISSION shRNA, Sigma)), 6 μg of envelope plasmid pMD2.G (Addgene; deposited by Dr. Didier Trono) and 6 μg of packaging plasmid pSPAX2 (Addgene; deposited by Dr. Didier Trono), using Lipofectamine 2000 Reagent (Invitrogen Life Technologies). After 12 h at 37 °C the medium was replaced with fresh DMEM containing 10% fetal bovine serum. Virus particles were harvested at 24 h and 48 h post-transfection. Adhered NSPCs were incubated with the lentivirus stock for 24 h in DMEM supplemented with 10% FBS and 12 additional hours in proliferation medium. Then, cells were cultured in suspension to allow neurosphere formation.

### 2.10 Immunoblotting

This protocol was performed as described in [1]. Briefly, cells were homogenized in lysis buffer (TRIS pH 7.6 1 M, 400 mM NaCl, 1 mM EDTA, 1 mM EGTA and 1% SDS) and samples heated at 95 °C for 15 min, sonicated and precleared by centrifugation. Primary antibodies used are detailed in Suppl. Table S1.

### 2.11 Quantitative real-time polymerase chain reaction (qRT-PCR)

Total RNA extraction and q-RT-PCR were done as detailed elsewhere [27]. Primer sequences are shown in Suppl. Table S2. All PCRs were performed in triplicate.

### 2.12 Statistical analysis

Data are presented as mean ± SEM. Statistical assessments of differences between groups were analyzed using GraphPad Prism 5 software by one or two-way ANOVA or unpaired Student's *t*-tests, assuming a normal distribution and equal variances as indicated in the legends.

## 3 Results

### 3.1 NRF2 deficiency impairs long term potentiation

**Fig. 1:**
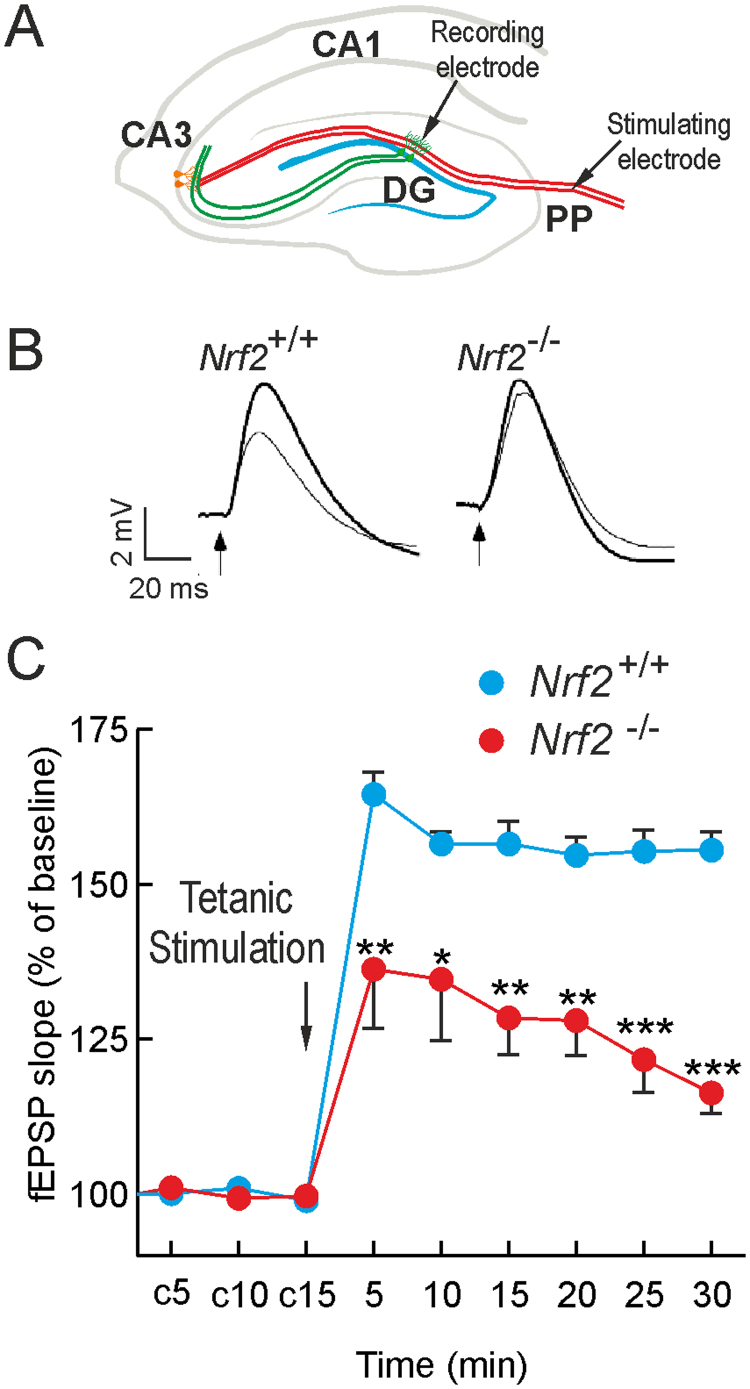
*NRF2 deficiency impairs long term potentiation.* A, upper draw points the positions of the stimulating electrode on the perforant pathway and recording electrode on the granule cell layer: DG, *dentate gyrus*; CA1 and CA3, *Cornu Ammonis* areas 1 and 3, respectively. B, representative responses recorded from *Nrf2*^+/+^ and *Nrf2*^-/-^ mice before (thin line) and after (thick line) high-frequency trains of tetanic stimulation. Calibration: 2 mV, 20 ms. C, LTP of field excitatory postsynaptic potential (fEPSP) in 6-month-old animals before and after tetanic stimulation of the perforant pathway. c5, c10, c15: baseline recordings before tetanic stimulation. Data are mean ± SEM (n = 6). Statistical analysis was performed with two-way ANOVA followed by Bonferroni post-hoc test. **p < 0.01, and ***p < 0.001 comparing *Nrf2*^-/-^ versus *Nrf2*^+/+^ group.

Adult *Nrf2*^+/+^ and *Nrf2*^-/-^ mice (6-months old) were examined electrophysiologically for hippocampal long term potentiation (LTP). The synaptic transmission of granule cells of the DG was recorded in response to perforant path (PP) stimulation (Fig. 1A). High frequency stimulation increased the field excitatory post-synaptic potential (fEPSP) in control *Nrf2*^+/+^ mice, but these values were strongly diminished in *Nrf2*^-/-^ mice (Fig. 1B-C), indicating for the first time that NRF2 is required for normal LTP. As the PP is dependent on the activity of excitatory granule neurons of the DG, and these cells are replaced by NSPCs from SGZ [28], we hypothesized that this might be due, at least in part, to impaired hippocampal neurogenesis in *Nrf2*^-/-^ mice.

### 3.2 The clonogenic and proliferative capacity of NSPCs from the SGZ is impaired in NRF2-deficient mice

**Fig. 2:**
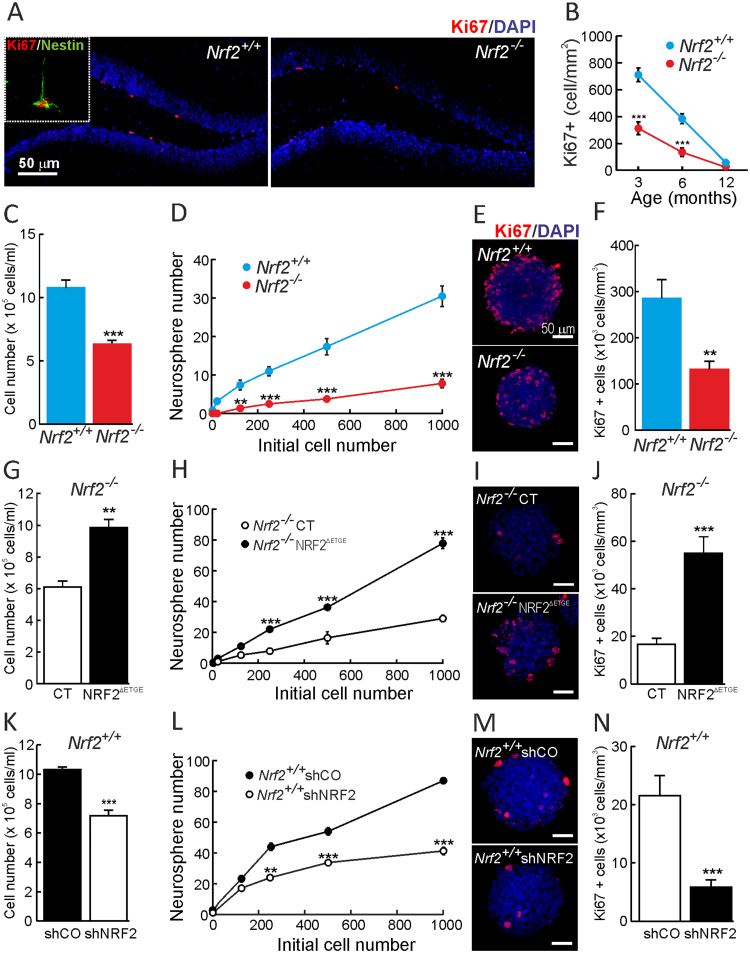
*Impaired proliferative and clonogenic capacity of NSPCs from Nrf2*^-/-^*mice.* A, confocal microscope images of Ki67 staining in the SGZ of 3-month-old mice. The green channel for Nestin staining has been removed except in the inset to allow easier visualization of proliferating cells. Nuclei were counterstained with DAPI. B, quantification of Ki67^+^ cells in 3-, 6- and 12-month-old mice. Data represent mean values ± SEM (n = 4). C, number of cells after 5 days in culture from an initial seeding of 20,000 NSPCs/ml derived from *Nrf2*^-/-^ vs. *Nrf2*^+/+^ 3-month-old mice. D, serial dilution assay of *Nrf2*^+/+^ and *Nrf2*^-/-^ cultures showing the number of neurospheres formed after 7 days (n = 5). E-F, immunofluorescence and quantification of Ki67^+^ cells, counterstained with DAPI, in neurospheres with similar size (n = 10). G-N, parallel analysis in *Nrf2*^-/-^-derived NSPCs infected with a control vector (CT) or a lentivirus expressing active NRF2^ΔETGE^ (G-J), and *Nrf2*^+/+^-derived NSPCs infected with control (shCO) and shNRF2 lentivirus (K-N). Data show mean values ± SEM. **p < 0.01, ***p < 0.001 according to a Student's *t*-test vs. either *Nrf2*^-/-^ (B, C, D, F) or the experimental groups (G,H, J, K, L and N).

**Supplemental Fig. 1:**
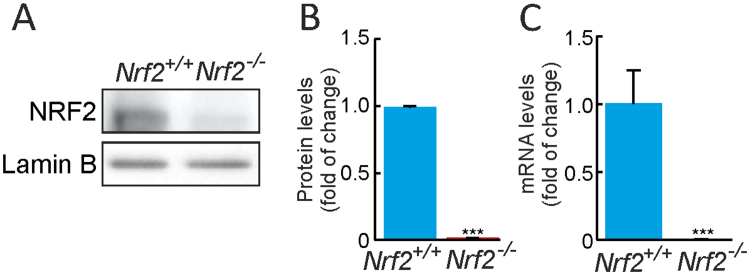
A, immunoblot analysis of NRF2 in *Nrf2*^+/+^ and *Nrf2*^-/-^ neurospheres. *Upper panel*, anti-NRF2 antibody showing the specific NRF2 band of ∼105 kDa. *Lower panel*, anti-Lamin B antibody to ensure similar protein load per lane. B, densitometric quantification of representative blots from A. Data are mean ± SEM (n = 3). Statistical analysis was performed with a Student's *t*-test. ***p < 0.001 vs. *Nrf2*^+/+^ group. C, relative expression of mRNA encoding NRF2 in *Nrf2*^+/+^ and *Nrf2*^-/-^ neurospheres normalized by β-actin mRNA levels. Data are mean ± SEM (n = 3). Statistical analysis was performed with a one-way ANOVA followed by Bonferroni post hoc test. ***p < 0.001 vs. *Nrf2*^+/+^ group.

**Supplemental Fig. 2:**
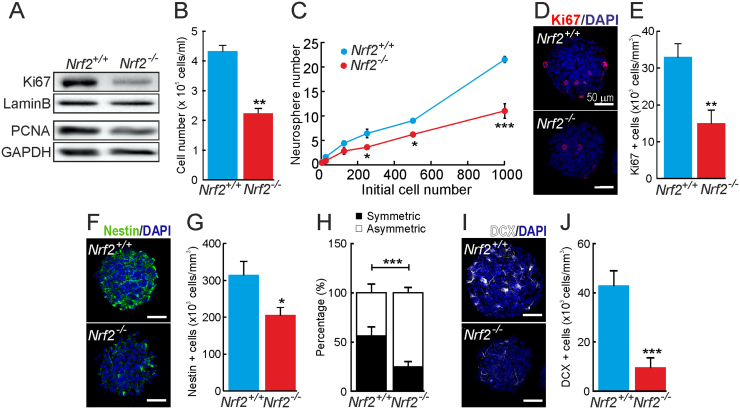
*Proliferative analysis and stem cells markers in neurospheres derived from newborn mice*. A, immunoblot analysis of proliferative markers Ki67 and PCNA. Respective Lamin B and GAPDH blots show similar amount of protein per lane. B, number of cells after 5 days in culture from an initial seeding of 20,000 NSPCs/ml derived from *Nrf2*^-/-^ vs. *Nrf2*^+/+^ newborn mice. C, serial dilution assay of newborn-derived *Nrf2*^+/+^ and *Nrf2*^-/-^ cultures showing the number of neurospheres formed after 7 days (n = 5). D-E, immunofluorescence and quantification of Ki67^+^ cells, counterstained with DAPI, in neurospheres with similar size (n = 10). F-G, Nestin immunofluorescence on neurospheres derived from 3-months-old mice (n = 10). H, quantification of symmetric and asymmetric divisions in cultures from newborn mice. I-J, DCX immunofluorescence in neurospheres derived from newborn mice (n = 10). Data represent mean values ± SEM. *p < 0.05, **p < 0.01 and ***p < 0.001 according to a Student's *t*-test vs. the *Nrf2*^-/-^ group.

**Supplemental Fig. 3:**
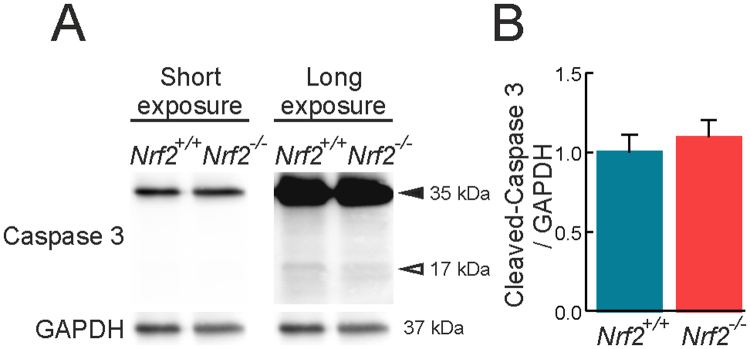
*Nrf2-deficiency does not increase apoptosis*. A, immunoblot analysis of Caspase 3 (35 kDa; filled arrow) and cleaved-Caspase 3 (17 kDa; empty arrow) in *Nrf2*^+/+^ and *Nrf2*^-/-^ neurospheres. Anti-GAPDH staining was performed to ensure similar protein load per lane. B, densitometric quantification of activated Caspase 3 from representative blots from A. Data are mean ± SEM (n = 3). Statistical analysis with a Student's *t*-test indicated non-significant differences.

We first confirmed by qRT-PCR and immunoblotting that NSPCs of the SGZ express NRF2 (Suppl. Fig. 1A-C) and performed immunostaining of the proliferative marker Ki67 in combination with the NSPC marker Nestin to evaluate the number of proliferative NSPCs in the SGZ (Fig. 2A-B). As expected both *Nrf2*^+/+^ and *Nrf2*^-/-^ mice exhibited a drastic reduction of proliferating cells with aging. However the number of proliferating NSPCs was ∼50% lower in *Nrf2*^-/-^ mice NSPCs. In order to compare proliferative and clonogenic capacities of NSPCs, we performed neurosphere assays from 3-months old mice. After 5 days in culture the number of cells in the *Nrf2*^-/-^ neurospheres was about ∼50% lower compared to *Nrf2*^+/+^ (Fig. 2C). Then, we found that NSPCs from *Nrf2*^-/-^ mice had a lower clonogenic capacity as determined by the number of neurospheres formed after seeding equal amounts of *Nrf2*^-/-^ and *Nrf2*^+/+^ NSPCs (Fig. 2D). In addition, Ki67 immunostaining also demonstrated a reduction in the number of proliferating cells in *Nrf2*^-/-^-derived neurospheres (Fig. 2E-F). In agreement with these results, immunoblot analyses indicated that two proliferative markers, Ki67 and PCNA, were reduced in *Nrf2*^-/-^-derived neurospheres (Suppl. Fig. 2A). Similar results were found in neurosphere cultures established from postnatal (P0-P4) mice (Suppl. Fig. 2B-E). In additional experiments, we analyzed caspase-3 cleavage as a measurement of cell death in the SGZ (data not shown) and neurospheres from both genotypes (Suppl. Fig. 3). We detected very low levels of cleaved caspase-3 by immunohistochemistry or immunoblot, suggesting that NSPCs are not undergoing apoptosis or they do it at undetectable level.

**Supplemental Fig. 4:**
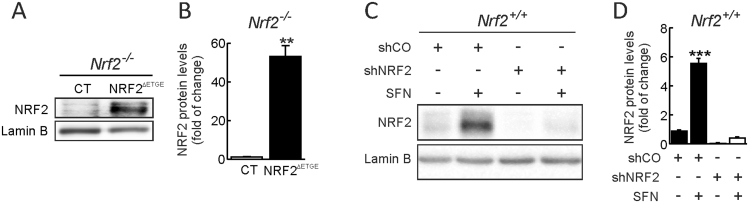
*Modulation of NRF2 protein levels by ectopic expression or shRNA knockdown*. A, immunoblot analysis of NRF2 protein levels in NSPCs infected with lentiviral empty vector (CT) or expressing active NRF2^ΔETGE^. *Upper panel*, anti-NRF2 antibody. *Lower panel*, anti-Lamin B antibody showing similar protein load per lane. B, densitometric quantification of representative blots from A. Data are mean ± SEM (n = 3). Statistical analysis was performed with a Student's *t*-test. **p < 0.01 vs. CT group. C, immunoblot analysis of NRF2 in *Nrf2*^+/+^ NSPCs transduced with control vector (shCO) or lenti-shNRF2 and treated with vehicle or sulforaphane (SFN, 15 µM, 6 h). *Upper panel*, anti-NRF2 antibody; *Lower panel*, anti-Lamin B. D, densitometric quantification of representative blots from C. Data are mean ± SEM (n = 3). Statistical analysis was performed with a one-way ANOVA followed by Bonferroni post hoc test. ***p < 0.001 vs. vehicle-treated shCO group.

In order to further analyze the involvement of NRF2 in these processes, we rescued NRF2 expression in *Nrf2*^-/-^ NSPCs with a lentiviral vector expressing a constitutively active mutant of NRF2 (NRF2^ΔETGE^) [26] (Suppl. Fig. 4A-B). Expression of NRF2 partially improved the clonogenic and proliferative capacity of *Nrf2*^-/-^ NSPCs, leading to increased number of neurospheres as well as of Ki67^+^ cells (Fig. 2G-J). Additionally, we silenced NRF2 with a lentiviral shRNA vector in *Nrf2*^+/+^ NSPCs. Evidence of NRF2-knockdown was provided after induction with the NRF2 activator sulforaphane (SFN) (Suppl. Fig. 4C-D). Knock-down of NRF2 led to a reduction in clonogenic and proliferating capacity of NSPCs (Fig. 2K-N). Taken together these results suggest a cell autonomous need of NRF2 to maintain the clonogenic and proliferative capacity of NSPC pool.

### 3.3 Age-related exhaustion of the NSPCs pool from the SGZ is exacerbated in NRF2-deficient mice

**Fig. 3:**
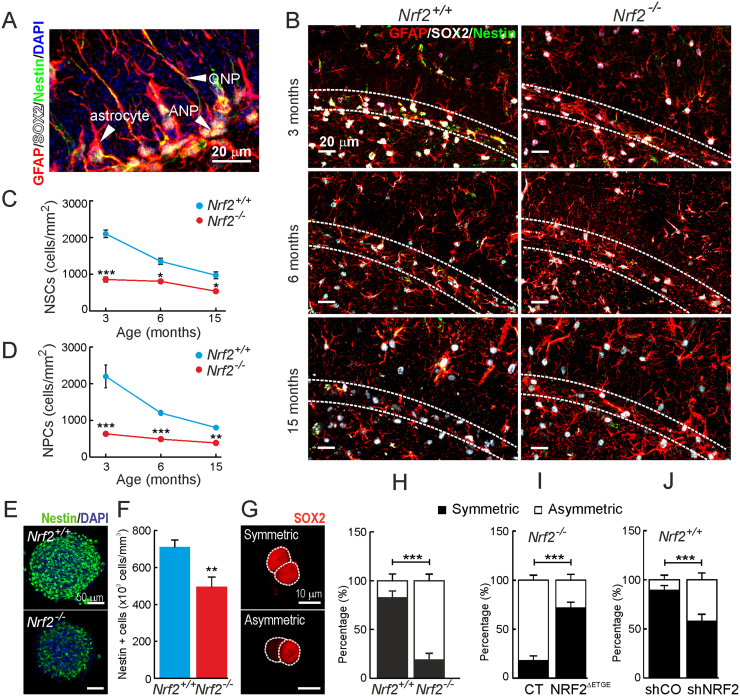
*NRF2-deficiency provokes a drop in the pool of QNPs and ANPs and an increase of asymmetric divisions of NSPCs*. A, confocal image of a SGZ immunostained GFAP/SOX2/Nestin. Arrows point a typical ANP, QNP and astrocyte (see text). B, same staining for 3- (Upper panel), 6- (middle panel) and 15- (low panel) month-old mice. C-D, quantification of QNPs (C) and ANPs (D) in 3-, 6- and 15-month-old mice (n = 3). E-F, nestin immunofluorescence on neurospheres derived from 3-month-old mice (n = 10). G-J, cell pair assay of NSPCs by SOX2 immunofluorescence. G, example of symmetric and asymmetric divisions. Quantification of symmetric and asymmetric divisions in cultures from 3-month-old (H) mice, *Nrf2*^-/-^ NSPCs infected with control or NRF2^ΔETGE^ expression lentivirus (I) and *Nrf2*^+/+^ NSPCs infected with control or shRNA lentivirus to knock-down NRF2 (J) (n = 60 pairs). Data show mean values ± SEM.*p < 0.05, **p < 0.01, ***p < 0.001 according to a student's *t*-test vs. either *Nrf2*^-/-^ (C, D, F and H) or the experimental groups (I and J).

We analyzed by immunostaining with Nestin, SOX2 and GFAP antibodies the number of quiescent neural progenitors (QNPs) and amplifying neural progenitors (ANPs), as these cell populations drive NSPCs towards mobilization and differentiation of neural lineages. QNPs were identified as Nestin^+^/SOX2^+^/GFAP^+^ cells and ANPs as Nestin^+^/SOX2^+^/GFAP^-^cells, and were distinguished morphologically: QNPs exhibit a non-branched long neurite across the granular cell layer while ANPs are located at the basis of the granular layer and do not present long neurites (Fig. 3A) [29]. The number of QNPs and ANPs in *Nrf2*^+/+^ mice decreased with aging (Fig. 3B-D). Thus at 15 months of age the number of QNPs and ANPs had decreased to ∼50% compared to 3 months of age. Importantly, in 3-month-old *Nrf2*^-/-^ mice the amount of these cells was already substantially decreased to ∼50%. Accordingly, the number of Nestin^+^ cells was decreased in *Nrf2*^-/-^ neurospheres from newborn (Suppl. Fig. 2F-G) and 3-month-old (Fig. 3E-F) mice.

As these results suggested impaired capacity of QNPs and ANPs to proliferate and differentiate, we further analyzed the number of symmetric and asymmetric divisions in cell culture following staining with SOX2 (Fig. 3G-J). Cells derived from symmetric divisions express SOX2 in both siblings, while in asymmetric divisions only one of the siblings expresses SOX2. As expected ∼75% of the *Nrf2*^+/+^ NSPCs derived from symmetric division. However in the *Nrf2*^-/-^ NSPCs symmetric divisions accounted for only ∼25% (Fig. 3H). In line with this, the *Nrf2*^-/-^ NSPCs from newborn mice exhibited an increment in asymmetric divisions as compared to control NSPCs (Suppl. Fig. 2H). These data were further confirmed by rescuing *Nrf2*^-/-^ NSPCs with the lentivirus-mediated expression of NRF2^ΔETGE^ (Fig. 3I). Moreover, knocking-down NRF2 in *Nrf2*^+/+^ NSPCs resulted in a reduction in symmetric divisions (Fig. 3J). Taken together, these results suggest that NRF2 deficiency limits the self-renewal capacity of the neurogenic niche of the SGZ.

### 3.4 Neuronal differentiation from NSPCs of the SGZ is impaired in NRF2-deficient mice

**Fig. 4:**
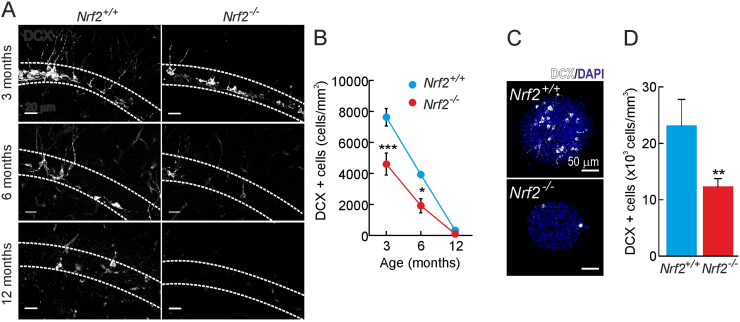
*NRF2-deficiency leads to a reduction of DCX+ cells.* A and B, DCX immunostaining in the SGZ of 3-, 6- and 12-month-old mice (n = 3). C-D, DCX immunofluorescence in neurospheres derived from 3-month-old mice (n = 10). Data represent mean values ± SEM. *p < 0.05, **p < 0.01 and ***p < 0.001 according to a Student's *t*-test vs. the *Nrf2*^-/-^ group.

We analyzed the capacity of the SGZ to produce new neurons using doublecortin (DCX) staining that specifically labels neuroblasts and immature neurons (Fig. 4A-B). In both *Nrf2*^+/+^ and *Nrf2*^-/-^ mice the amount of DCX^+^ cells decreased gradually to ∼10% at 12 months of age but the SGZ of *Nrf2*^-/-^ mice presented a lower capacity to generate these neuronal progenitors already at 3 and 6 months of age. These results were also analyzed in neurospheres and again the amount of DCX^+^ cells was substantially decreased to ∼20% in newborn (Suppl. Fig. 2H-I) and ∼50% in 3-month-old *Nrf2*^-/-^ mice (Fig. 4C-D).

**Fig. 5:**
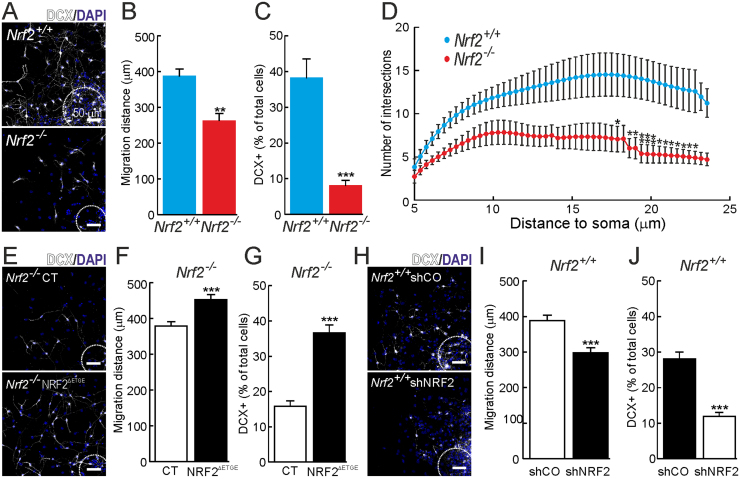
*Role of Nrf2 in the neuronal differentiation of NSPCs.* A, DCX and DAPI staining of neurospheres from 3-months old mice grown under differentiation conditions. B, measurement of the cell migration as determined by distance from the neurosphere edge (dotted line) to DAPI stained nuclei (n = 40). C, quantification of DCX^+^ cells from neurospheres shown in A (n = 10). D, sholl analysis of the neurons in the differentiated neurospheres derived from 3-months old mice (n = 12). E. DCX and DAPI staining of *Nrf2*^-/-^ neurospheres rescued by lentiviral expression of NRF2^ΔETGE^. F, measurement of their cell migration distance (n = 40). G, quantification of DCX^+^ cells (n = 10). H-J, parallel analysis in *Nrf2*^+/+^ neurospheres infected with control of shRNA lentivirus to knockdown NRF2. Data represent mean values ± SEM. *p < 0.05, **p < 0.01 and ***p < 0.001 according to a Student's *t*-test vs. the *Nrf2*^-/-^ group (B, C and D) or the experimental groups (F, G, I and J).

**Supplemental Fig. 5:**
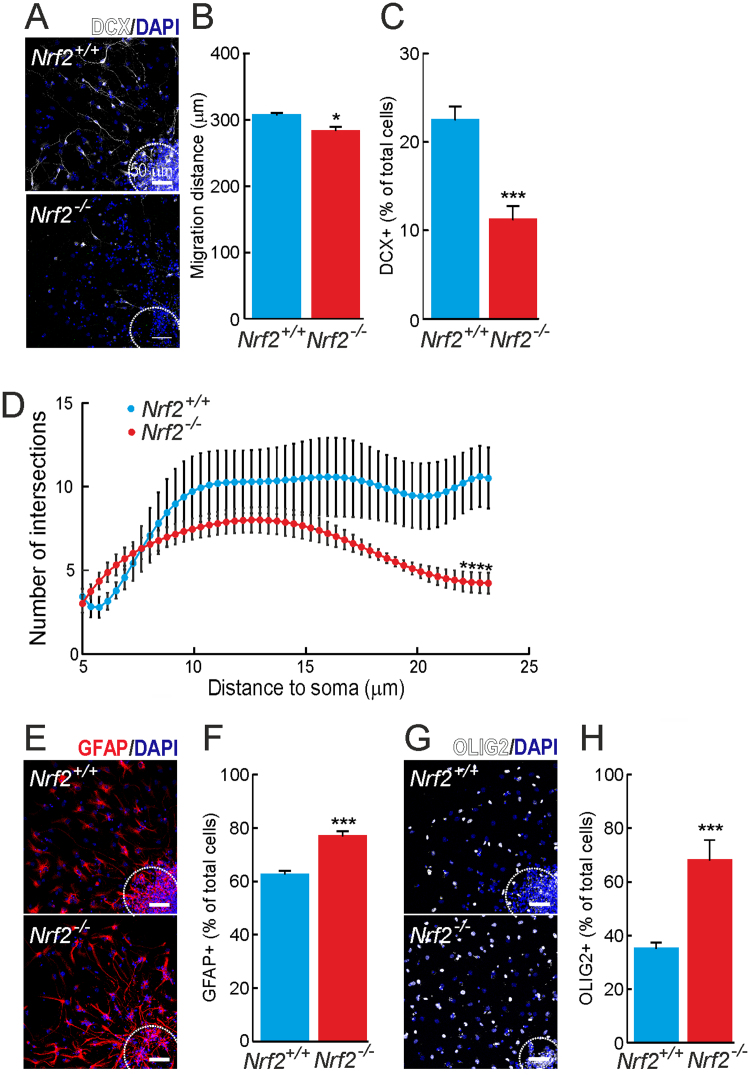
*Neuronal and glial differentiation in neurospheres derived from newborn mice*. A, DCX and DAPI staining of neurospheres from newborn mice grown under differentiation conditions. B, measurement of the cell migration as determined by distance from the neurosphere edge (dotted line) to DAPI stained nuclei (n = 40). C, quantification of DCX^+^ cells from neurospheres shown in A (n = 10). D, sholl analysis of the neurons in the differentiated neurospheres derived from newborn mice (n = 12). E-F, astroglial differentiation analyzed by GFAP immunostaining in the neurospheres derived from newborn mice (n = 10). G-H, Oligodendrocyte differentiation analyzed in vitro using Olig2 staining in the differentiated neurospheres derived from newborn mice (n = 10). Data shown mean values ± SEM. *p < 0.05, **p < 0.01, ***p < 0.001 according to a Student's *t*-test vs. either *Nrf2*^-/-^ group.

Next, we analyzed the differentiation potential of the NSPCs. When plated on poly-d-lysine and grown in the absence of growth factors NSPCs migrate away from the neurosphere core and spread around forming a carpet of cells differentiated to neurons, astrocytes and oligodendrocytes [30]. First, following DCX and DAPI staining, we noted a decrease in the migration distance of cells from newborn (Suppl. Fig. 5A-B) and, more notoriously, from 3-month-old (Fig. 5A-B) *Nrf2*^-/-^ mice. The migration distance was improved by reintroduction of NRF2 expression in *Nrf2*^-/-^ cultures (Fig. 5E-F), and worsened by knock-down of NRF2 in *Nrf2*^+/+^ cultures (Fig. 5H-I). DCX staining in the differentiating cultures indicated impairment in the neuronal differentiation of *Nrf2*^-/-^ neurospheres at birth (Suppl. Fig. 5C) and at 3 months of age (Fig. 5C). Moreover, rescue of NRF2 expression in *Nrf2*^-/-^ cultures led to enhanced neuronal differentiation (Fig. 5G). On the other hand, silencing NRF2 in *Nrf2*^+/+^ neurospheres reduced significantly the percentage of DCX^+^ cells (Fig. 5J). To analyze the maturation of these cells and their capacity to interact with other neurons, we performed a Sholl analysis, that measures the branching complexity, in newborn (Suppl. Fig. 5D) and 3-month-old (Fig. 5D) mice. DCX^+^ cells from *Nrf2*^-/-^ mice showed a decrease in the number of neurite crossings suggesting a reduced maturation compared to those of *Nrf2*^+/+^ mice. All these data suggest that NRF2 is not only important to maintain the NSPCs pool but also to support a proper neuronal differentiation.

### 3.5 NRF2-deficiency impairs the neuron/glia differentiation balance

**Fig. 6:**
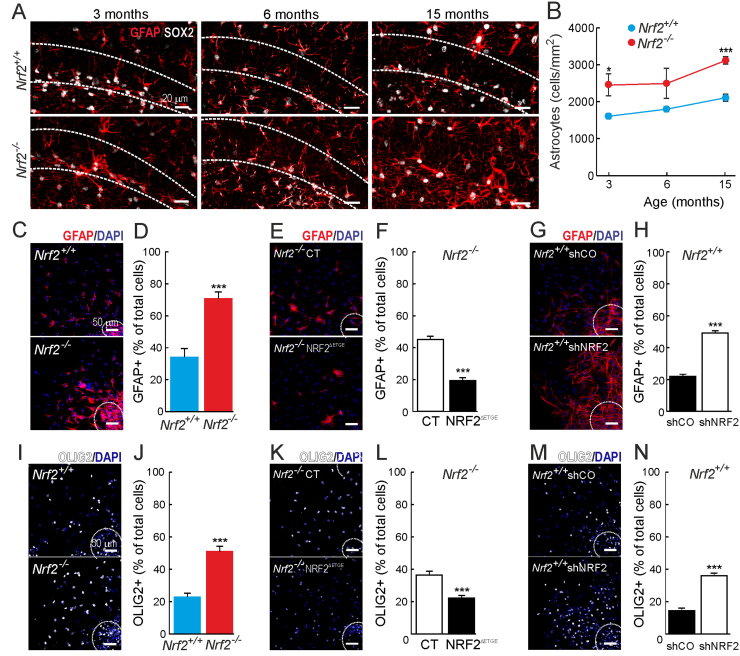
*Nrf2-deficiency leads to an increment in non-neuronal differentiation.* A and B, astrocyte differentiation analyzed in the SGZ of 3-, 6- and 15-months old mice by GFAP/SOX2 immunostaining (n = 3). C-H, astroglial differentiation analyzed by GFAP immunostaining in the neurospheres derived from 3-month-old mice (A and B), *Nrf2*^-/-^ neurospheres derived from control and NRF2^ΔETGE^ expressing NCSs (C and D), and *Nrf2*^+/+^ neurospheres derived from control NRF2-knock-down NSPCs (E and F) (n = 10). I-N., oligodendrocyte differentiation analyzed in vitro using Olig2 staining in the differentiated neurospheres derived from 3-month-old mice (I and J), *Nrf2*^-/-^ neurospheres derived from control and NRF2^ΔETGE^ expressing NCSs (K and L), and *Nrf2*^+/+^ neurospheres derived from control NRF2-knock-down NSPCs (M and N) (n = 10). Data shown mean values ± SEM. *p < 0.05, **p < 0.01, ***p < 0.001 according to a Student's *t*-test vs. either *Nrf2*^-/-^ group (B, D, J, L and N) or the experimental groups (F, H, P and R).

Astrocyte differentiation in the SGZ was analyzed by GFAP and SOX2 staining (Fig. 6A-B). The *Nrf2*^-/-^ mice exhibited a growing increase in astrocyte abundance at the SGZ for 3, 6 and 15 months of age. When we analyzed the astroglial differentiation in vitro, using GFAP staining, we noted an increase in the number of astrocytes in *Nrf2*^-/-^ vs *Nrf2*^+/+^ cultures, being 20% higher in newborn (Suppl. Fig. 5E-F) and 40% higher in 3-month-old-derived neurospheres (Fig. 6C-D). The increase in astrocytes was also observed *Nrf2*^+/+^ cultures when we silenced NRF2 expression (Fig. 6G-H). Furthermore, recovering NRF2 expression in *Nrf2*^-/-^ cultures resulted in a reduction to 20% of GFAP^+^ cells (Fig. 6E-F). Oligodendrocyte differentiation was analyzed with OLIG2 staining. *Nrf2*^-/-^ neurospheres from newborn (Suppl. Fig. 5G-H) and 3-month-old mice (Fig. 6I-J) exhibited ∼2-folds increase in the number of oligodendrocytes. In line with these results, reintroduction of NRF2 in *Nrf2*^-/-^ NSPCs reduced significantly the number of OLIG2-positive cells (Fig. 6K-L) while NRF2 silencing in *Nrf2*^+/+^ cultures resulted in an enrichment in oligodendrocytes under differentiating conditions (Fig. 6M-N). These results could not be replicated in mice (data not shown), in agreement with reports suggesting that NSPCs from the SGZ might not generate oligodendrocytes in vivo [31], [32]. All these measurements indicate that NRF2 is a key regulator of the balance between neuronal and glial differentiation.

## 4 Discussion

Here we report for the first time the role of NRF2 in mobilization and differentiation of NSPCs in the SGZ, a neurogenic niche that participates in neurogenesis of the adult hippocampus and therefore has crucial implications in preservation of cognitive functions. SGZ NSPCs appear to have a crucial role in the replacement of new neurons in granule cell layer [33]. These adult-born neurons synaptically integrate in the pre-existing hippocampal neural circuitry and participate in LTP, which plays a role in learning and memory [13], [34], [35]. In turn, depletion of hippocampal neurogenesis results in LTP loss [36], [37].

It has been reported that local induction of oxidative stress during mobilization of neural precursors can occur given the evidence that oxidized DNA and lipids are present in the SGZ of adult rodents [38]. Although the authors did not analyze NRF2 directly, a bioinformatics analysis based on previous transcriptomics data sets [39], [40] did show changes in the levels of several antioxidant enzymes whose expression is regulated by NRF2. We could not analyze NRF2 protein levels by immunohistochemistry in the SGZ (data not shown) and, in fact, NRF2 levels were almost undetectable by immunoblotting in NSPCs isolated from the SGZ (Suppl. Fig. 1) and SVZ (data not shown). However, these cells responded to the NRF2 activator SFN indicating that although low, NRF2 is expressed under basal conditions and responds to environmental inducers. To avoid off-target effects of NRF2 activating drugs, we used a genetic approach based on lentiviral reintroduction of NRF2 in NSCs. Ectopic expression of NRF2 rescued otherwise NRF2-deficient NSPCs to cloning and proliferative values, similar to those of wild type NSPCs. Our results are in agreement with a previous study showing that up-regulation of NRF2 ameliorated amyloid β-mediated neural stem cell death [41]. This study also demonstrated that neuronal differentiation of NSPCs is enhanced by NRF2 overexpression [41]. Additionally, we found that decreasing or increasing NRF2 expression by genetic means was sufficient to significantly suppress or rejuvenate the neurogenic niche of the SGZ. Furthermore, SGZ NSPCs from either new born or 3 month-old *Nrf2*^-/-^ mice exhibited substantially compromised proliferation and neuronal differentiation. Our results are in line with a previous study showing that a highly reduced intracellular redox state promotes proliferation and survival, whereas a highly oxidized state results in greater differentiation and cell death [42].

In order to understand how NRF2 controls the fate of NSPCs, we used the cell pair assay that indicates whether NCS divisions lead to identical siblings with proliferating capacity (symmetric division) or to two different cells, one retaining proliferation capacity and the other entering a differentiation program (asymmetric division) [25]. The current model of mobilization of the NSPC pool establishes that a NSC progenitor will divide symmetrically for several generations to maintain the progenitor pool. At the same time, a certain number of asymmetric divisions will lead to a supply of neurons as well as astrocytes to accommodate specific demands. The last division leads to differentiation of both siblings into astrocytes [29]. The fact that the NSPC pool was exhausted more quickly in the *Nrf2*^-/-^ mice suggested that either cell-autonomous alterations or micro-environmental influence leads to the mobilization and terminal differentiation of these neural progenitors. Consistent with this hypothesis, we found an inverse correlation between Ki67-stained progenitors (Fig. 2) and GFAP-stained astrocytes (Fig. 6). Moreover, in the cell pair assay, we found that NRF2 deficiency resulted in an increase of asymmetric divisions, leading to the reduction in the number of progenitors. These findings suggest that the exhaustion of the NSPC pool in the *Nrf2*^-/-^ mice is due to an increase in asymmetric divisions, together with imbalance in the cell fate to increase the number of astrocytes and reduce neurons. This hypothesis is consistent with the well reported observation that NRF2 is required to counterbalance the high levels of intracellular ROS of highly proliferating cells [43], [44]. Furthermore, our study also connects the observed pattern of decline in NSPC function with cognitive impairment.

These results identify for the first time NRF2 as an important factor to prevent the decline in the NSPC pool of the SGZ, during aging, and have important implications towards understanding fundamental aspects of NSPC biology and promising therapeutic interventions in disorders involving hippocampal function.
