# Supplementary material for: Transcription factor NRF2 controls the fate of neural stem cells in the subgranular zone of the hippocampus
Source: Redox Biol. 2017 Jun 27;13:393–401. doi: 10.1016/j.redox.2017.06.010 (PMC5493838; doi:10.1016/j.redox.2017.06.010)
Supplement: Supplementary file 1 — Supplementary material [file mmc1.docx]

**Supplemental Table S1.** List of primary antibodies for immunofluorescence (IF) and western blot (WB).

| Antibody | Source | Catalog Number | Dilution |
| --- | --- | --- | --- |
| Caspase 3 | Cell Signaling | 9662 | 1:1000 (WB) |
| DCX | Santa Cruz Biotechnology, INC. | sc-8066 | 1:200 (IF) |
| GAPDH | Merck Millipore | CB1001 | 1:15000 (WB) |
| GFAP | Dako | Z0334 | 1:500 (IF) |
| GFAP | Sigma | G3893 | 1:200 (IF) |
| Ki67 | Abcam | 16667 | 1:2000 (IF)  1:5000 (WB) |
| LaminB | Santa Cruz Biotechnology, INC. | sc-6217 | 1:2000 (WB) |
| Nestin | Abcam | Ab11306 | 1:200 (IF) |
| Nrf2 | Homemade [26] |  | 1:2000 (WB) |
| Olig2 | Chemicon International | AB9610 | 1:200 (IF) |
| PCNA | Abcam | Ab2426 | 1:2000 (WB) |
| SOX2 | R&D Systems | AF2018 | 1:200 (IF)  1:2000 (WB) |

**Supplemental Table S2.** Primers sequences.

| Gene | Forward Sequence 5´-3´ | Reverse Sequence 5´-3´ |
| --- | --- | --- |
| *β-Actin* | TCCTTCCTGGGCATGGAG | AGGAGGAGCAATGATCTTGATCTT |
| *Nfe2l2 (Nrf2)* | CCCGAAGCACGCTGAAGGCA | ccaggcggtgggtctCCGTA |
